# Assessing the Appropriateness of Formulations on the WHO Model List of Essential Medicines for Children: Development of a Paediatric Quality Target Product Profile Tool

**DOI:** 10.3390/pharmaceutics14030473

**Published:** 2022-02-22

**Authors:** Jennifer Walsh, Tiziana Masini, Benedikt D. Huttner, Lorenzo Moja, Martina Penazzato, Bernadette Cappello

**Affiliations:** 1 Jenny Walsh Consulting Ltd., BioCity Nottingham, Nottingham NG1 1GF, UK; 2 Independent Researcher, 55041 Lucca, Italy; tizianamasini@gmail.com; 3 WHO Department of Health Products, Policy and Standards, World Health Organization, 1211 Geneva, Switzerland; bhuttner@who.int (B.D.H.); mojal@who.int (L.M.); cappellob@who.int (B.C.); 4 WHO Research for Health Department, World Health Organization, 1211 Geneva, Switzerland; penazzatom@who.int

**Keywords:** essential medicines, paediatric, formulations, public health, age appropriate, WHO model list of essential medicines for children

## Abstract

The World Health Organization’s Model List of Essential Medicines for Children (EMLc) presents a list of the most efficacious, safe, and cost-effective medicines for priority conditions, intended for use in children up to 12 years of age. However, gaps in global availability and use of age-appropriate formulations of medicines for children still exist. To address these shortcomings, a comprehensive analysis of the appropriateness of formulations of essential medicines for children is being undertaken through the Global Accelerator for Paediatric Formulations (GAP-f) network, a WHO network launched in 2020 to respond to the paediatric treatment gap. This article describes the development and application of a paediatric Quality Target Product Profile (pQTPP) tool by WHO, to retrospectively evaluate the paediatric age-appropriateness of formulations on the EMLc and identify potential formulation gaps, to inform the review of the EMLc in 2023. A combination of paediatric-centric and global health-focused attributes and targets were defined, taking into consideration regulatory agency paediatric development guidelines and literature sources, and a qualitative scoring system was developed and tested. Example evaluations of paracetamol and clofazimine are provided, illustrating the tool’s use. The assessment of EMLc formulations is ongoing and shortcomings and gaps in EMLc formulations have already been identified. The pQTTP tool may also be applied to national lists and prospectively when designing new paediatric formulations.

## 1. Introduction

Despite regulatory incentives, and increasing efforts and resources dedicated by researchers and public-private partnerships to address and promote the development of formulations for children [1,2,3], there is still a global paucity of age-appropriate formulations of paediatric medicines to treat and prevent a variety of conditions, especially in low- and middle-income countries (LMICs) [4,5,6,7,8]. This may result in the need to manipulate formulations intended for adults when treating children, for example, tablet splitting or crushing, which may affect drug exposure and lead to inaccurate dosing and potentially sub-optimal treatment or adverse events [4,9,10]. Formulation manipulation may also substantially complicate dosing and negatively impact treatment tolerability, patient acceptability, and long-term adherence, which may even prevent the use of a medicine in children outright [11].

Following the resolution at the 69th World Health Assembly on promoting innovation and access to quality, safe, efficacious, and affordable medicines for children [12], the Global Accelerator for Paediatric Formulations (GAP-f) was created as a World Health Organization (WHO)-hosted platform to provide a sustainable mechanism dedicated to ensuring that the most-needed optimal paediatric formulations are developed and made available to children in a timely manner [13] (© World Health Organization [2022]. All rights reserved. The World Health Organization has granted the Publisher permission for the reproduction of this article.). GAP-f builds on and complements several initiatives that have emerged to focus efforts to deliver on this global commitment and scale-up activities to ensure that age-appropriate formulations are available for children [14,15,16].

In 2007, WHO published its first Model List of Essential Medicines for Children (EMLc) [17] and has since then updated the list every two years, with the most recent one published in October 2021 [18]. The EMLc is an evidence-based list of medicines to satisfy the priority health care needs of children up to 12 years of age. Essential medicines are intended “to be available in functioning health systems at all times, in appropriate dosage forms, of assured quality and at prices individuals and health systems can afford”. The EMLc is used as a guide by many countries in the development and updating of their national essential medicines lists (NEMLs) and paediatric formularies.

Under the framework of GAP-f, WHO is currently carrying out a comprehensive and thorough review of the EMLc to inform the next update of the list in 2023. This assessment will identify formulations to be proposed for potential addition given their therapeutic utility in children, as well as identify formulations to be proposed for deletion because they are not appropriate. This project will also help to identify gaps to inform additional research and development (R&D) activities to fill urgent unmet formulation needs for the paediatric population.

To facilitate the review of the paediatric age-appropriateness of formulations on the EMLc, WHO designed and applied an assessment tool. According to the International Conference on Harmonisation of technical Requirements for Registration of Pharmaceuticals for Human Use (ICH) Q8 (R2), “all medicinal products should be designed to meet patients’ needs and the intended product performance” [19]. The development and application of a Quality Target Product Profile (QTPP) is a well-recognised tool within pharmaceutical development. The QTPP forms the basis of the design of a drug product and considers various product attributes including for example route of administration, dosage form, dose strength, and container closure, as well as product attributes that impact pharmacokinetic properties and the quality of the drug product [16]. The use of a paediatric-focussed QTPP whereby additional attributes of key relevance to paediatric patients are included has been recommended to facilitate the development of new age-appropriate formulations [20].

Although QTPPs are usually used prospectively in the design of new pharmaceutical products, the development and utilisation of a new tool, based on a paediatric-specific QTPP (pQTPP), which also included attributes focusing on the needs of LMICs, was conceptualised by WHO in 2020 to retrospectively evaluate existing formulations on the EMLc. This article describes the development of the pQTPP tool, including the design of a scoring system that allows the user to identify gaps in terms of product attributes or specific needs of the paediatric population. Two examples illustrating the application and utility of the pQTPP tool to medicines listed in the EMLc are also provided.

## 2. Materials and Methods

The first step in the process for designing the tool was to identify paediatric-centric attributes to be included in the pQTPP. An initial list was collated based upon recommendations and considerations for developing paediatric formulations discussed in regulatory agency paediatric development guidelines and literature sources [17,21,22,23,24,25]. In addition, the specific needs of, and challenges associated with medicine supply in LMICs were considered, including, for example, high humidity and temperatures, along with rudimentary and fragmented storage and transportation facilities which often lack temperature control [26]. Hence, medicine stability in non-temperate climates is an important attribute as well as the primary packaging, which should ideally be compact with a small bulk footprint, light in weight, and sufficiently robust to withstand transportation in rural areas [23]. Targets for each of the proposed attributes were then defined based on regulatory guidance documents, taking into consideration the needs of paediatric patients as well as LMICs [18].

In order to evaluate the paediatric-age appropriateness of the EMLc formulations, a scoring method was required. A qualitative scoring system was proposed to assess each medicine dosage form against the target for each predefined attribute [27]. Several scoring and risk assessment approaches were considered, for example, the application of quality risk management tools (ICH Q9) [28] and the use of an evidence to decision framework [29], the applicability and utility of which were tested using an example EMLc formulation (i.e., amoxicillin powder for oral liquid).

A prototype tool was developed and tested independently by two individuals (JW and TM), using two different dosage form types as example formulations on the EMLc (amoxicillin powder for oral liquid and dapsone tablets) and the attribute list, target attributes, and scoring system were simplified as a result. Between January and April 2021, a series of virtual seminars were held with various WHO experts and partners to share the aims of the project, describe the proposed tool, and seek feedback and agreement. These experts included WHO disease area focal points for tuberculosis, HIV, malaria, hepatitis, mental health and behavioural disorders, childhood cancer, neurological diseases, and neglected tropical diseases, as well as key GAP-f stakeholders including Medicines for Malaria Venture, PENTA Foundation, St George’s University, UK, and the Bill and Melinda Gates Foundation. These conversations led to further modifications of the tool, to ensure that all key aspects relevant for a broad variety of disease areas would be taken into account.

The final tool was pilot tested with specific formulations of medicines for hepatitis C and leprosy, representing recently developed and old formulations respectively. Two individuals (JW and TM) compared their independent evaluations for consistency and the results of these assessments were then shared with and corroborated by WHO disease area experts. A tool user guide was developed in parallel, which included attribute scoring criteria, to facilitate the use of the tool and promote consistency in evaluation of formulations on the EMLc.

## 3. Results and Discussion

### 3.1. Development of the Tool: Paediatric QTPP Attributes and Targets

Although global paediatric regulations define the paediatric population as being aged from birth to less than 16 or 18 years [30], the EMLc is intended for use in children up to the age of 12 years; therefore, the tool was designed to focus on this age group. Regulatory agency guidelines on paediatric pharmaceutical development have provided recommendations regarding the need for dose flexibility, patient acceptability, and excipient safety, as well the requirement to consider method of medicine administration and the use of appropriate measuring devices [18] and these aspects have been extensively discussed in the literature [1,5,19,21,31,32,33,34,35,36,37,38,39]. It is of key importance that paediatric patients can easily be administered the required dose and hence a formulation should allow the required flexibility of dosing according to age, body weight, or surface area, as appropriate. The acceptability of a medicine is determined by the characteristics of the product and the user [18]. For the purposes of the pQTPP tool, pharmaceutical characteristics such as swallowability, palatability, size of dose (number of tablets or capsules, volume of liquid, quantity of granules, ointment, or cream), and frequency of dosing were considered for the acceptability attribute. It is recognised that the need to modify the medicine prior to administration (for example mixing with food or dilution), and the requirement for and ease of use of an administration device can also impact acceptability. However, these aspects were included as a separate attribute under “administration”.

Excipients may be considered as being inert ingredients; however, they can have different effects in children compared to adults and were therefore included as an attribute within the tool. Colouring agents, flavours, preservatives, and sugars and sweeteners can be of particular concern [18,40]. For example, the preservative benzoic acid and benzoates should not be used in neonates because accumulation may occur in these patients due their immature metabolising enzymes, which can lead to an increase in bilirubinaemia and potentially kernicterus [41]. In addition, polyol sweeteners such as sorbitol can have osmotic laxative effects which may be more pronounced in children compared to adults and can also impact the bioavailability of concomitant medicines [42].

As discussed above, the provision of age-appropriate medicines in LMICs faces additional challenges, for example, the climatic conditions which may require products to be stable in high temperature and high humidity storage conditions and the need for low bulk footprint and easily transported packaging due to fragmented supply chains. Hence, shelf life, storage conditions, and packaging were added to the pQTPP tool attribute list. Furthermore, affordability is an important consideration for LMICs, and a “patient access” attribute was added to take this into account. The regulatory status of the drug products and whether product licences had been granted by a Stringent Regulatory Authority (SRA) was added to provide a measure of quality of available products. In particular, approvals by the United States Food and Drug Administration (USFDA), European Medicines Agency (EMA), United Kingdom Medicines and Healthcare Regulatory Authority (MHRA), and Australia Therapeutic Goods Administration (TGA) were assessed. In addition, interrogation of regulatory documentation, including the Summary of Product Characteristics (SmPC) or label, provides valuable information on, for example, approved posology, excipients, storage conditions, shelf life, and primary packaging, all of which are required to facilitate the formulation evaluation process.

During the pilot testing of the tool, it was noted that some formulations, notably solid oral dosage forms such as conventional tablets and capsules intended for swallowing, would be scored as “moderate risk” for the acceptability attribute, as they were considered acceptable for older children, but not acceptable for young children who could not swallow them, i.e., unsuitable for some patients. The acceptability attribute was therefore split into two age sub-sets; birth to 5 years and 6 to 12 years, to enable greater differentiation in acceptability according to patient age but without adding additional complexity. It is considered that patients aged from approximately 6 years are able to swallow tablets, although it is recognised that this depends on the ability of the child and dimensions of the dosage form [18,36,43]. Indeed, it has been reported that tablets are well accepted in children aged 6 years and above and may be preferred by their caregivers for medicine administration to school age children [4,9,10].

In addition, during discussions with WHO disease area focal point and GAP-f partners, it became clear that the acquisition of robust and consistent data on affordability would be very challenging, since this may not be readily available in the public domain and also depends on numerous factors, including country-specific procurement policies. This attribute was therefore removed from the final tool; formulation assessors would have the opportunity to add comments to their reviews, including, for example, if the formulation technology utilised was complex and therefore the product could potentially have a high cost of goods.

The final paediatric QTPP (pQTPP) attributes and their respective targets agreed upon by key stakeholders are shown in Table 1.

### 3.2. Development of the Tool: Qualitative Scoring System

During the testing of potential scoring approaches, it became clear that a simple, qualitative scoring system would meet the needs of the project and enable the identification of potentially unsuitable formulations on the EMLc, as well as paediatric formulation gaps.

A simple, qualitative scoring system (Table 2) was devised to minimize complexity whereby the result of each attribute for each formulation was compared to the target and rated as follows:

- Low risk/no issues; meets target.
- Moderate risk/issues; partially meets target.
- High risk/issues; does not meet target.

Each rating was allocated a score and colour, based on a “traffic-light” system, to enable a heat map of each formulation to be visualised. Low risk attributes were scored 3 (green); moderate risk attributes were scored 2 (yellow); and high-risk attributes were scored 1 (red). A score of 0 (grey) was allocated for attributes where there was no or insufficient information to conduct an evaluation.

When developing medicines for children, the selection of a paediatric dosage form should consider the comparative benefits and risks of different pharmaceutical design options to help determine the relative advantages and disadvantages of each [44]. The weighting or prioritisation of some attributes over others can be applied and was considered for the pQTPP tool but not progressed since such a weighting system would need to be developed and applied on a case-by-case basis. Hence a more generic qualitative approach was deemed to be most appropriate for the aims of the project.

Feedback from WHO disease area focal point and GAP-f partners acquired during demonstrations of the tool supported the use of this scoring system.

### 3.3. Application of the Tool: Formulation Evaluation Process

Regulatory agency approved SmPCs or labelling was used as the primary source of information for medicines, from which details such as indicated age range, posology, dose administration instructions, excipients, primary packaging, shelf life, and storage conditions could be extracted. The results of these searches were recorded in the tool according to each attribute and compared against the target. For formulations where more than one product licence is available, an overview of the results for each attribute were recorded. The dose flexibility of the formulation was evaluated considering the required posology and concentration or dose strength of the medicine. Where dose information was provided on a mg/kg basis, required doses according to age were estimated using 50th percentile figures recorded on WHO weight-for-age charts (https://www.who.int/tools/child-growth-standards/standards/weight-for-age, accessed on 13 December 2021). Acceptability was evaluated using literature sources of information [45]. The potential risk of the inclusion of an excipient within a formulation depends on various factors including its dose, route of administration, the age of the patient, duration of treatment, and indication. Since precise information on the full quantitative composition of formulations is not available in the public domain, this attribute was evaluated by considering the presence of potential excipients of concern within the formulations, as well as their quantity if reported and their potential function. For example, the inclusion of ethanol or propylene glycol as a solvent within a liquid formulation would be considered a much higher risk compared to their inclusion at a very low concentration within a flavouring. Where quantitative information on an excipient was provided, the total daily intake on a mg per Kg body weight basis was estimated and compared with available safety information, for example, WHO and European Food Safety Authority (EFSA) derived acceptable daily intakes (ADIs).

For the administration of liquids, the ability to measure doses accurately is required to reduce the risk of dosing errors. This is especially important in young patients where low dose volumes may be required; volumes less than 0.1 mL were considered to be unacceptable [5]. However, appropriately sized syringes should be used and it is recognised that in a domiciliary setting, untrained or inexperienced caregivers may have greater difficulty in identifying and measuring correct doses compared to trained healthcare professionals.

Table 2 shows scoring criteria developed to facilitate consistency in evaluations.

### 3.4. Application of the Tool: Example Evaluations

Evaluations for paracetamol (analgesic, anti-pyretic) and clofazimine (for treatment of leprosy) are provided below to illustrate the use of the tool. Paracetamol was selected since several formulations of this medicine are listed on the EMLc, whilst clofazimine, used for the treatment of leprosy, was selected to illustrate issues that may exist regarding available formulations to treat neglected tropical diseases.

#### 3.4.1. Paracetamol

Paracetamol is widely used globally and is listed in the EMLc as follows:

*Section 2.1 Non-opioids and non-steroidal anti-inflammatory medicines:*

Oral liquid; 120 mg/5 mL; 125 mg/5 mL.

Suppository; 100 mg.

Tablet; 100 mg to 500 mg.

*Section 7.1 For treatment of acute migraine attack:*

Oral liquid; 120 mg/5 mL; 125 mg/5 mL.

Tablet; 300 mg to 500 mg.

Evaluations for each paracetamol dosage form are shown in Tables S1–S3 (supplementary information) and a summary of the evaluations across dosage forms, including assigned scoring, is shown in Table 3.

Paracetamol (acetaminophen) is commonly used for the management of mild-to moderate pain and fever in children and is considered as first line therapy for these indications. In addition, it is the drug of choice for the treatment of mild-to-moderate pain in neonates, although care is required in patients suffering from dehydration or malnutrition due to potential risk of overdose and toxicity [48]. Numerous paracetamol oral liquid and tablet formulations are available and the current recommended dose is 10 to 15 mg/kg every 4 to 6 h (up to 60 mg/kg/day), although some differences in dosing recommendations were noted between different countries during the assessment, as previously reported [45]. As shown in Table 3, evaluation of the different paracetamol formulations using the pQTTP tool has shown that none fully meet all the target attributes. Indeed, although the tablets contain acceptable excipients and have a suitable shelf life, storage conditions, and packaging, they have limited dose flexibility and are only considered acceptable for patients who can swallow them. In contrast, the oral liquids are easy to swallow and have high flexibility of dosing but generally contain preservatives, sweeteners, and flavouring which may be problematic in some patients, and some variants contain sucrose and/or colour which should only be used in paediatric products if necessary. Furthermore, the oral liquids are less favourable from a stability, shelf life, and packaging perspective compared to solid oral dosage forms. Paracetamol suppositories offer an alternative option for dosing young children from approximately 3 months of age. It should be noted that there may be cultural barriers to suppository administration, and they may be more commonly administered in a healthcare setting [49]. The stability, shelf life, and packaging of paracetamol suppositories appear to be generally more favourable for LMICs compared to oral liquids, although dose flexibility is limited; the addition of higher dose suppositories may reduce the number of suppositories required per dose for older children. Similarly, the addition of a higher strength formulation of paracetamol liquid (e.g., 250 mg/5 mL) may facilitate dosing to older children who have difficulty swallowing tablets, since it would reduce the volume required per dose; children aged from 9 or 10 years may require dose volumes greater than 10 mL of the current strength oral liquids. However, the availability of multiple strengths of the same medicine may potentially lead to mis-dosing due to the selection and administration of the incorrect strength product, as well as additional complexity to the supply chain.

Overall, a combination of paracetamol tablets, oral liquid, and suppositories provide suitable dosage form options for patients aged from approximately 2–3 months (depending on the product licence) up to 12 years, and even adulthood, although none are ideal, and they each have different advantages and disadvantages. During the review of these EMLc-listed paracetamol formulations, other paracetamol formulations were identified, one of which was paracetamol dispersible tablets, in 100 mg, 120 mg, and 250 mg dose strengths. Dispersible tablets are dispersed in a small volume of water prior to administration and are thus easy to swallow and appropriate for young children. Although not fully reviewed, paracetamol dispersible tablets are likely to have favourable excipients, stability, and primary packaging and may therefore be a potential alternative to oral liquids [23], and so should be considered for addition to the EMLc. Indeed, flexible solid oral dosage forms such as orodispersible tablets or tablets that can be used for the preparation of oral liquids, for example, dispersible or soluble tablets, have been recommended for use in LMICs, although they may not be suitable when precise dose titration is required [22].

#### 3.4.2. Clofazimine

Clofazimine is used to treat leprosy as part of a multidrug therapy including rifampicin and dapsone and is also used as a second-line medicine to treat multidrug-/rifampicin-resistant (MDR/RR-) tuberculosis (TB). It is listed in the EMLc as follows:

*Section 6.2.4—Antileprosy medicines:*

Capsule; 50 mg; 100 mg.

*Section 6.2.5—Antituberculosis medicines (Complementary List):*

Solid oral dosage form; 50 mg; 100 mg.

The assessment shown in Tables S4 and S5 (Supplementary Materials) includes a review of the appropriateness of listed formulations for both leprosy and TB. However, the analysis reported here focuses on the use of clofazimine capsules in children only for leprosy and is summarized in Table 4 below.

Over 200,000 leprosy cases were registered globally in 2019, of which almost 15,000 were reported to be in children aged below 14 years, resulting in an incidence of 7.9 per million child population. One of the targets of WHO’s Global Leprosy Strategy is a 90% reduction in the rate per million children of new leprosy cases by 2030 [50].

The standard WHO-recommended treatment regimen for leprosy includes a three-drug regimen of rifampicin, dapsone, and clofazimine for all leprosy patients, with a duration of treatment of 6 and 12 months for paucibacillary and multibacillary leprosy, respectively. For leprosy, clofazimine (as 50 mg or 100 mg soft-gel capsules) is provided free of charge to countries and national leprosy programmes through WHO, as part of multidrug regimens together with rifampicin and dapsone in standard blister packs. However, access to clofazimine outside the WHO donation programme might be hindered by the lack of clofazimine registration by any SRA. Indeed, clofazimine capsules have not been approved for the treatment of tuberculosis or leprosy by any of the SRAs reviewed in the context of this project and were therefore assigned the lowest score for the attribute corresponding to the registration status. This holds true also for clofazimine tablets.

For children aged 10–14 years, clofazimine should be administered at a dose of 150 mg once a month and 50 mg on alternate days, while for children aged below 10 years or below 40 kg, clofazimine should be given at a dose of 100 mg once a month and 50 mg twice weekly [51]. This dosing schedule in children below 10 years or age (or below 40 kg) is the only option available given that the lowest clofazimine dosage strength corresponds to 50 mg and soft-gel capsules cannot be opened and the contents administered by dispersing in water, due to the extremely hydrophobic nature of the active pharmaceutical ingredient. While on one hand the low dose flexibility (scored as only partially meeting the target) does not impact drug exposure given the drug’s long elimination half-life, on the other hand acceptability for young children who are unable to swallow capsules is very limited. Therefore, patient acceptability for children below 6 years of age was scored as not meeting the target, while acceptability for children aged between 6 and 12 years was scored as meeting the target after taking into consideration the relatively small size of clofazimine capsules (round 7 mm).

Soft-gel capsules are not ideal for resource-limited settings, given their sensitivity to humidity and high temperatures. The WHO-supplied blister packs are provided in humidity-resistant containers, which protect clofazimine capsules from moisture. Although the capsules have a long shelf life, they must be stored below 25 °C. In addition, the capsules contain some excipients of potential concern for paediatric patients including, for example, propylene glycol and parabens.

Even though clofazimine tablets (50 mg, 100 mg) are now available, with a better stability profile than capsules (i.e., they are not sensitive to humidity and thus are preferred for LMICs), they are still less widely available compared to capsules and they are not dispersible, (Table S5); therefore, a truly child-friendly formulation of clofazimine is still to be developed.

Our assessment highlights a major gap to dose children aged below 6 years, for which a suitable, acceptable paediatric formulation of clofazimine is not yet marketed. This is confirmed by clinicians’ experience even in high-resource settings who struggle to dose young children, especially if only soft-gel capsules are available (irrespective of whether they aim for daily or less frequent dosing), given the difficulty to manipulate the contents of the capsule [52].

### 3.5. Application of the Tool: General Considerations

The application of the pQTPP tool to paracetamol and clofazimine has identified a number of gaps and issues regarding the availability of age-appropriate formulations for these two medicines which are currently listed on the EMLc. In addition, it has shown that it is hard to balance all the needs of paediatric patients in a global setting and that more than one dosage form is often required to meet the needs of the entire paediatric population. Orubu et al. (2021) evaluated the age-appropriateness of enteral (oral or rectal) dosage forms on the EMLc based on swallowability (where appropriate) and the ability to administer the correct doses, according to ICH paediatric age-subsets [53]. They concluded that most recommended enteral essential medicines in EMLc 2011 and 2019 were not age-appropriate for children <6 years and that unsuitable medicines must be manipulated before administration leading to concerns regarding safety and efficacy. Our initial evaluations appear to be broadly in line with their findings in that some formulations are not age-appropriate for young patients. However, the study by Orubu et al. did not consider the additional needs of LMICs related to stability in non-temperate climates and supply chain. Therefore, although an oral liquid may be scored as highly acceptable for young patients (green, based on our qualitative scoring system), it would receive a lower score (yellow or red) for the stability and supply chain attributes and may also be less favourable from an excipient perspective. As discussed above, the use of flexible solid oral dosage forms such as orodispersible or dispersible tablets may be appropriate for paediatric patients in LMICs [22]. It should be noted that although dispersible tablets are easy to prepare, some reluctance to reconstitute them has been reported, and oral liquids are still favoured by young children. This might be partly due to patient and caregiver unfamiliarity with dispersible tablets and therefore education and engagement with local communities is recommended [6,54]. In addition, the use of multi-particulates (granules) or mini-tablets may be suitable since they are considered to be easy to swallow, may be administered with a beverage or soft food vehicle, are likely to be more stable than oral liquids, and offer some flexibility of dosing [42]. Indeed, there are examples of emerging medicines for children for the treatment of HIV and hepatitis C utilising these formulation approaches [55,56,57,58], and there appears to be an overall trend towards preservative-free, taste-masked solid oral dosage forms for paediatric patients globally [59].

Although paediatric medicines should be developed for the intended patient population, where a specific paediatric formulation is not available, the dispersion of conventional tablets or the contents of a hard gelatin capsule in water or other vehicle could provide a suitable strategy to meet stability and swallowability needs, particularly in LMICs, to allow the dosing of children who cannot swallow whole tablets, assuming compatibility with the vehicle and the taste of the dispersion and dose is acceptable. For example, imatinib solid oral dosage form which is listed in the EMLc for treatment of leukaemias and gastrointestinal stromal tumours, is available as scored tablets and hard gelatin capsules, both of which according to their SmPCs may be administered via dispersion in water or apple juice. The formulations are available in 400 mg and 100 mg dose strengths, which provide suitable doses for older children and adolescents and patients aged 6 years and above, respectively. In addition, according to the SmPC, the scored tablets may be split and divided into equal doses, i.e., 200 mg or 50 mg. Therefore, an alternative option for administration is available for patients unable to swallow the solid oral dosage form whole. However, the taste of the drug when dispersed is not known and dose flexibility is limited, and a bespoke paediatric formulation would be preferable.

The assessment of formulations on the EMLc is ongoing. The pQTPP tool evaluation results for each medicine will be shared with the relevant WHO disease area focal experts, including recommendations for formulations that should be considered for removal and those for potential addition. The results will be scrutinized by the relevant experts on a case-by-case basis, to determine whether or not applications for amendments to the EMLc should be submitted for consideration at the 24th meeting of the Expert Committee on the Selection and Use of Essential Medicines, to be held in 2023. It should be noted that although a formulation may exhibit several “high risk” (red score) attributes, further appraisal of its use and alternatives may not lead to its removal from the EMLc, if the benefit of its inclusion outweighs any potential formulation risks. Any formulation gaps identified, i.e., where an age-appropriate formulation does not appear to be available or exist, will be highlighted and shared with the wider paediatric research community to stimulate and encourage the development of new products.

It is recognised that there are some limitations to the pQTPP tool. For example, multiple formulations of the same drug dosage form may be available, and it may not be practical to review them all. Thus, an overview result may need to be recorded for some attributes. A similar approach is required for excipients as stated above, due to the lack of publicly available information on specific quantities of each formulation excipient. Despite these challenges, the pQTPP tool has been shown to be able to identify EMLc formulation gaps and thus fulfil its purpose.

## 4. Conclusions

The EMLc promotes access to affordable, safe, and effective medicines for children. A new pQTPP tool has been successfully developed by WHO for the retrospective evaluation of formulations on the EMLc which includes attributes reflecting essential features of paediatric formulations for global use and an easy to use scoring system. The tool presented will aid a comprehensive and thorough review of the 2021 EMLc to evaluate appropriateness of the currently listed formulations and identify gaps that can inform the submission of proposed additions in the 2023 update of the list. Although the full impact of the assessment tool may not be determined until the completion of the EMLc 2021 review and publication of the updated EMLc in 2023, the two example evaluations have clearly illustrated its utility. The tool may also be applied prospectively for the development of new paediatric medicines including the re-purposing of off-patent drugs for paediatric use.

A copy of the pQTPP is available in Supplementary Materials.

## Figures and Tables

**Table 1:** Paediatric Quality Target Product Profile attributes and targets.

Attribute	Target	Comments
Target population (age)	Entire age range 0 to ≤12 years	Target population is for WHO EMLc. Ideally the product should be suitable from birth although patient population age will depend upon the medicine and indication. The drug product may be restricted to a paediatric age sub-set. If no age or weight limits are listed, it is assumed the product is intended for 0 to ≤12 years.
Dose and dose flexibility	Defined paediatric dose range and dose increments	Product concentration/strength and format should allow correct and flexible dosing, according to patient age, weight, or body surface area. Dose banding may be possible.
Patient acceptability	Acceptable for the proposed patient population	Dosage form must be suitable for use in the proposed paediatric population. Different dosage forms may be required for different age groups. Depends on many factors including route of administration, dosage form, and patient/caregiver characteristics (including age, disease, ability).
Excipient safety	Excipients with acceptable safety profile for the proposed patient population.	Excipient benefit versus risk should be considered if product excipients are listed (e.g., on label). Where excipient details are unavailable, potential excipient risks associated with dosage form should be considered (e.g., preservatives, sweeteners, surfactants, co-solvents in liquids).
Administration considerations	Required doses can be easily and accurately administered, with minimal preparation	Evaluate according to setting (e.g., domiciliary versus healthcare facility) and characteristics of individual administering the product. Administration device (if required) should be readily available and appropriate for the intended use. Multiple dilutions should be avoided. Guidance on compatible administration vehicle(s)/diluents and storage time (if required) should be available. Proposed dosing vehicles should be readily available. Accuracy of splitting scored tablets (if relevant) to be considered.
Stability, storage conditions and primary packaging material	Stable for 2 years minimum under long term storage conditions (ICH). Packaging suitable for hospital and/or home use, easy to use and unambiguous.	Global climatic conditions should be considered, including for in-use stability if applicable. Refrigerated (2–8 °C) and freezer storage is less favourable. Primary packaging should ideally be light weight, portable, and with child-resistant closure. If specific information on pack and shelf life is unavailable, potential pack options and stability according to dosage form/formulation type should be considered.
Registration status	Positive opinion or approved by a Stringent Regulatory Authority	Regulatory status and potential registration strategy (if required) to be considered. Prior approval can facilitate further/subsequent license approvals and WHO pre-qualification.

**Table 2:** Paediatric Quality Target Product Profile tool scoring criteria.

Attribute	Considerations for Scoring		
	High Risk/Issues; Does Not Meet Target Score = 1	Moderate Risk/Issues; Partially Meets Target Score = 2	Low Risk/No Issues; Meets Target Score = 3
Target population (age) (0 to ≤12 years) ^1^	Not suitable for all or the majority of patients aged less than 12 years	Suitable for most of the API indicated paediatric population	Suitable from birth Suitable for the API indicated age range
Dose and dose flexibility ^2^	Lack of or poor dose flexibility Not able to administer the required doses without manipulation	Some limited dose flexibility, (e.g., limited dose strengths available). Not able to administer the required doses to some patients.	High dose flexibility. Able to easily measure and administer the required doses to all patients.
Patient acceptability ^3^ 0–5 years	Unacceptable for this age range, e.g., conventional tablets/capsules. Anticipated to have strongly aversive taste, painful injection, etc.	Some concerns re. acceptability in this age range, e.g., poor palatability, frequent dosing, formulation unsuitable for some patients.	Acceptable for this age range.
Patient acceptability ^3^ 6–12 years			
Excipient safety ^4^	Contains several excipients of potential or known concerns.	Contains 1 or 2 excipients of potential concern.	Contains excipients which generally have an acceptable safety profile.
Administration Considerations ^5^	Complex manipulation required, e.g., reconstitution with fixed volume of vehicle (domiciliary use), multiple dilutions (HCP and domiciliary use). Complex administration device/procedure (HCP and domiciliary use).	Some manipulation required (e.g., food mixing) or measurement of dose required (domiciliary use). Some manipulation required (e.g., food mixing, reconstitution with fixed volume of vehicle) or measurement of dose required (HCP use).	No manipulation or measurement required (domiciliary use). No manipulation required, easy to measure and administer required doses (HCP use).
Stability, storage conditions, primary packaging material ^6^	Requires freezer or refrigerated storage. Less than 18 months shelf life. Bulky/heavy packaging. Complex packaging design.	May be stored under room temperature conditions, ^7^ but constituted product requires refrigerated storage. Requires protection from moisture. Less than 2 years shelf life.	May be stored under room temperature conditions. ^7^ Minimum 2-year shelf life. Light packaging with low bulk footprint. Simple packaging design.
Registration status	Not approved by any Regulatory Authorities and no approvals anticipated.	Approved by a Regulatory Authority with maturity level 3 and above [46,47]. ^8^ Approval by Stringent Regulatory Authority anticipated.	Approved by at least one Stringent Regulatory Authority.

^1^ The lowest indicated or recommended age should be considered. Minimum age may be older than from birth. For example, if the condition is only prevalent or possible to diagnose from 3 years, minimum target age = 3 years. ^2^ Strength or concentration should allow the required doses to be accurately and easily administered. Tablet splitting may be permitted if supported by the product license. Dose banding may be possible. ^3^ Score according to age group. Numerous factors involved—an overall score should be applied. Excipient considerations should be excluded and scored separately. Frequent dosing is mitigated by short-term use. ^4^ Excipient safety will depend on the route of administration. Neonates are more vulnerable to excipient “adverse effects” compared to older children. ^5^ For administration of the required dose. Need to consider setting, availability of device (if required), complexity of process, potential for mis-dosing or dosing errors. ^6^ If shelf life is not listed in label, consider dosage form/formulation type, handling, required storage conditions, and packaging type. ^7^ Defined here as 20–25 °C (USP <659> Packaging and Storage Requirements defines controlled room temperature as 20–25 °C). ^8^ Maturity level 3 is defined as “stable, well-functioning and integrated regulatory systems”; maturity level 4 is defined as “regulatory systems operating at advanced level of performance and continuous improvement”.

**Table 3:** pQTPP summary for paracetamol formulations listed in the WHO Essential Medicines List for children.

Attribute/Dosage Form	Paracetamol Tablet	Paracetamol Liquid	Paracetamol Suppository
Target population	2	3	3
	Only suitable for those able to swallow tablets	Suitable for whole population	Suitable for whole population
Dose and dose flexibility	2	3	2
	Limited dose flexibility	High dose flexibility	Limited dose flexibility
Patient acceptability (0–5 years)	1	3	3
	Not acceptable for patients unable to swallow tablets or requiring a low dose	Easy to swallow	Accepted by young patients (need to consider culture)
Patient acceptability (6–12 years)	3	2	2
	Acceptable assuming patient can swallow a tablet	Older children may require high volumes (15–20 mL)	Less accepted by older children and may need multiple suppositories (need to consider culture)
Excipient safety	3	1	3
	Excipients generally have acceptable safety profile	Contains several excipients of concern	Excipients generally have acceptable safety profile
Administration considerations	3	2	2
	No manipulation required for tablet	Doses must be measured with device	Some caregivers/patients may have difficulty with correct insertion, some patients may experience discomfort
Stability, storage conditions and primary packaging material	3	2	3
	Sufficient shelf life, easy to transport	Acceptable shelf life but bottles bulky to transport	Likely acceptable shelf life, easy to transport
Registration status	3	3	3
	Approved by Stringent Regulatory Authorities	Approved by Stringent Regulatory Authorities	Approved by Stringent Regulatory Authorities

**Table 4:** pQTPP summary for clofazimine formulations listed in the WHO Essential Medicines List for children in the Antileprosy medicines section (Section 6.2.4).

Attribute/Dosage Form	Clofazimine Capsules (50 mg, 100 mg)
Target population	2
	Only suitable for those able to swallow tablets
Dose and dose flexibility	2
	Limited dose flexibility. Dosing with available strengths requires administration on alt days for children <10 y
Patient acceptability (0–5 years)	1
	Not acceptable for patients unable to swallow capsules
Patient acceptability (6–12 y)	3
	Acceptable assuming patient can swallow a capsule and considering the relatively small size of the clofazimine capsule
Excipient safety	2
	Capsules contain some excipients of potential concern
Administration considerations	3
	No manipulation required for capsules
Stability, storage conditions, and primary packaging material	2
	Acceptable shelf life; preparation supplied in a humidity-resistant container, but capsules should be stored below 25 °C
Registration status	1
	Not approved by Stringent Regulatory Authorities

## Data Availability

Not applicable.

## Supplementary Materials

The following are available online at https://www.mdpi.com/article/10.3390/pharmaceutics14030473/s1, Table S1: Evaluation of Paracetamol Oral tablets (100 mg to 500 mg); Table S2: Evaluation of Paracetamol Oral Liquid (120 mg/5 mL; 125 mg/5 mL); Table S3: Evaluation of Paracetamol Suppository (100 mg); Table S4: Evaluation of Clofazimine (soft-gel capsules, 50 mg and 100 mg); Table S5: evaluation of Clofazimine (tablets, 50 mg and 100 mg). The paediatric QTPP tool is also available as an Excel spreadsheet.
