# Supplementary material for: Assessing the Appropriateness of Formulations on the WHO Model List of Essential Medicines for Children: Development of a Paediatric Quality Target Product Profile Tool
Source: Pharmaceutics. 2022 Feb 22;14(3):473. doi: 10.3390/pharmaceutics14030473 (PMC8950931; doi:10.3390/pharmaceutics14030473)
Supplement: Supplementary file 1 [file pharmaceutics-14-00473-s001.zip › pharmaceutics-1557362--supplementary.pdf]

pharmaceutics

Supplementary Materials: Assessing the Appropriateness of Formulations on the WHO Model List of Essential Medicines for Children: Development of a Paediatric Quality Target Product Profile Tool

Jennifer Walsh, Tiziana Masini, Benedikt D Huttner, Lorenzo Moja, Martina Penazzato and Bernadette Cappello [3]

Table S1. Evaluation of Paracetamol Oral Tablets (100 mg to 500 mg).

Attribute	Result	Comparison to Target and Risk Assessment	Score	Additional considerations/information
**Target population (age)**	Minimum age and posology depends on PL and market, e.g., (UK) 4Kg (2-3 months) 60mg (up to 2 doses); 3-6 months 60mg (up to 4x/day); 6-24 months 120mg (up to 4x/day); 2-4 years 180mg (up to 4x/day); 4-6 years 240mg (up to 4x/day); 6-8 years 250mg (up to 4x/day); 8-10 years 375mg (up to 4x/day); 10-12 years 500mg (up to 4x/day); (USA) 2.7-5Kg (≤3 months) 40mg (up to 5x/24h); 5-8Kg (4-11 months) 80mg (up to 5x/24h); 8-11Kg (12-23 months) 120mg (up to 5x/24h); 11-16Kg (2-3 years) 160mg (up to 5x/24h); 16-21Kg (4-5 years) 240mg (up to 5x/24h); 22-27Kg (6-8years) 320mg (up to 5x/24h); 27-32Kg (9-10 years) 400mg (up to 5x/24h); 33-43Kg (11 years) 480mg (up to 5x/24h).	Partially meets target/moderate risk. Only suitable for patients able to swallow tablets (dimensions not known). *However, dispersible tablets may be suitable for patients aged from approx. 6 months depending on PL.*	2	Minimum indicated age depends on PL. Oral liquid would be an alternative for younger patients requiring a lower dose.
**Dose and dose flexibility**	Minimum dose possible depends on market, PL and availability of products. Some tablets have score lines and so may be able to dose e.g., 250mg. Limited dose flexibility.	Partially meets target/moderate risk. Limited dose flexibility, able to dose patients who can swallow tablets (e.g., from approx. 6 years).	2	Oral liquid or other alternative dosage form should be used in patients unable to swallow tablets and/or requiring a lower dose.
**Patient acceptability: 0-5 years**	Unacceptable for patients unable to swallow tablets and/or requiring a lower dose. Potentially frequent dosing (up to 5x day dosing).	Does not meet target/high risk for patients unable to swallow tablets whole (depending on tablet dimensions), and young patients requiring a lower dose than tablet dose units available. 4-5x daily dosing	1	Use of an oral liquid or other alternative dosage form should be considered for young patients.

Attribute	Result	Comparison to Target and Risk Assessment	Score	Additional considerations/information
		but short-term use. *Dispersible tablets easy to swallow, especially for younger patients and may be suitable from 6-12 months.*		
**Patient acceptability: 6-12 years**	Acceptable for patients able to swallow tablet, although potential frequent dosing (up to 5x day dosing).	Meets target/low risk assuming patient can swallow tablet whole. Tablet dose units available acceptable for proposed dosing.	3	Older children and adolescents may prefer tablets.
**Excipient safety**	Contain standard tabletting excipients.	Meets target/low risk. Excipients generally have a suitable safety profile.	3	
**Administration considerations**	Tablets are intended to be swallowed whole. No manipulation required, although those with a score line could potentially be split for dosing. *Dispersible tablets require dispersal in a small volume of liquid for administration.*	Meets target/low risk. No manipulation required (unless break tablet along score line), although some patients may have difficulty in swallowing the tablets whole. Not able to administer doses required for young patients.	3	Low risk of dosing errors for older children, although some patients may have difficulty in swallowing the tablets whole. Not able to administer doses required for young patients.
**Stability, storage conditions and primary packaging material**	Shelf life depends on product but appears to be 3-4 years. Storage 20-25°C. Primary pack is light weight (HDPE bottle or blisters). *Dispersible tablets require protection from moisture.*	Meets target/low risk - likely sufficient shelf life and product easily transported (generally light weight).	3	
**Registration status**	Numerous licences available, including in USA, UK, Europe, Australia- generic and branded. Also available OTC. Variable dosing recommendations across the globe	Meets target/low risk. Approved by stringent regulatory authorities.	3	Drug product strengths and dosing recommendations appear to vary.

Table S2. Evaluation of Paracetamol Oral liquid (120 mg/5 mL; 125 mg/5 mL).

Attribute	Result	Comparison to Target and Risk Assessment	Score	Additional considerations/information
**Target population (age)**	Minimum age and posology depends on PL and market, e.g., (UK) 4Kg (2-3 months) 60mg (up to 2 doses); 3-6 months 60mg (up to 4x/day); 6-24 months 120mg (up to 4x/day); 2-4 years 180mg (up to 4x/day); 4-6 years 240mg (up to 4x/day); 6-8 years 250mg (up to 4x/day); 8-10 years 375mg (up to 4x/day); 10-12 years 500mg (up to 4x/day); (USA) 2.7-5Kg (≤3 months) 40mg (up to 5x/24h); 5-8Kg (4-11 months) 80mg (up to 5x/24h); 8-11Kg (12-23 months) 120mg (up to 5x/24h); 11-16Kg (2-3 years) 160mg (up to 5x/24h); 16-21Kg (4-5 years) 240mg (up to 5x/24h); 22-27Kg (6-8years) 320mg (up to 5x/24h); 27-32Kg (9-10 years) 400mg (up to 5x/24h); 33-43Kg (11 years) 480mg (up to 5x/24h).	Low risk/no issues; meets target. API indicated from 2 months - product strengths available are suitable for the proposed paediatric population according to labelled dosing.	3	High dosing flexibility possible.
**Dose and dose flexibility**	High dose flexibility, able to easily measure the required doses in increments of 2.5mL (depending on product). Older patients, e.g., from ~ 9 years may require dose volumes greater than 10 mL.	Low risk/no issues; meets target. Able to easily dose patients aged from 2 months up to 12 years.	3	A higher concentration suspension (i.e., 160/5mL and/or 250mg/5mL) would be more suitable for dosing older patients (from approx. 8/9 years).
**Patient acceptability: 0-5 years**	Oral liquids easy to swallow. Product contains sweeteners and flavours to mask taste of API. Potentially frequent dosing.	Low risk/no issues; meets target. Assume acceptable palatability due to sweetener and flavourings. 4-5x daily dosing but short-term use.	3	
**Patient acceptability: 6-12 years**	Oral liquids easy to swallow but older patients may require high volumes per dose (e.g., 15-20 mL).	Moderate risk/issues; partially meets target due for older children requiring high dose volumes. Assume acceptable palatability due to sweetener and flavourings. 4-5x daily dosing but short-term use.	2	Older patients could take a tablet if preferred. Higher concentration suspension would reduce dose volume.
**Excipient safety**	Varies depending on formulation/PL. e.g, may contain sucrose, glycerol, and/or sorbitol solution. Also contain preservatives (some include methyl, ethyl and propyl	High risk/issues; does not meet target due to the presence of several excipients of concern.	1	Generally short- term use.

Attribute	Result	Comparison to Target and Risk Assessment	Score	Additional considerations/information
	paraben), surfactant, sweetener, flavouring. Many contain colourants.			
**Administration considerations**	Doses required to be measured, dosing spoon or oral syringe could be used.	Moderate risk/issues; partially meets target. Doses must be measured using a measuring device (e.g., dosing spoon). Volumes larger than 5mL require more than one spoonful to be administered	2	Tablets might be more suitable for older patients, or use of a higher concentration suspension (i.e., 160mg/5mL or 250mg/5mL).
**Stability, storage conditions and primary packaging material**	Shelf life generally 2-3 years, in-use not listed. Storage conditions- room temperature (20-25°C), do not store above 25°C. Primary pack is PET or glass bottle	Moderate risk/issues; partially meets target. Shelf life acceptable but bottles, especially glass, may be bulky to transport.	2	
**Registration status**	Numerous licences available, including in USA, UK, Europe, Australia- generic and branded. Other strength products also available in some markets (160mg/5mL, 250mg/5mL, 100mg/mL). Posology appears to vary globally.	Meets target/low risk. Approved by stringent regulatory authorities.	3	

Table S3. Evaluation of Paracetamol Suppository (100 mg).

Attribute	Result	Comparison to Target and Risk Assessment	Score	Additional considerations/information
**Target population (age)**	Minimum age and posology depend on PL and market, e.g., (UK) 5Kg (3 months) 60mg, 10kg (1 year) 120 or 125mg, 20Kg (5-6 years) 250mg, 40Kg (12 years) 500mg (all up to 4x/24h); (USA) 2-4 years 160mg, 4-6 years 240mg, 6-9 years, 320mg, 9-11 years 320-400mg, 11-12 years, 320-480mg (all up to 5x/24h) Dosage in < 2 years individualised.	Low risk/no issues; meets target. Suppositories are suitable from 1 month, minimum age depends on PL and product strength, e.g., from 2 months post immunisation (60mg).	3	Suppositories useful when the oral route cannot be used e.g., due to vomiting, unconsciousness etc.
**Dose and dose flexibility**	Very limited dose flexibility as currently only increments of 100mg possible - it is not recommended to cut suppositories. Older children would require several suppositories per dose.	Moderate risk/issues; partially meets target. Limited dose flexibility, able to dose from approx. 1 year with 100mg product.	2	The addition of other suppository strengths to EMLc would increase dosing flexibility.
**Patient acceptability: 0-5 years**	Suppositories are suitable for the paediatric population, although may not be acceptable to some caregivers due to cultural concerns and unfamiliarity. Potentially frequent dosing but short term.	Low risk/ no issues; meets target. Accepted by younger patients. 4x daily dosing but short- term use.	3	
**Patient acceptability: 6-12 years**	Suppositories are suitable for the paediatric population, although may not be acceptable to some caregivers/patients due to cultural concerns and unfamiliarity. Potentially frequent dosing but short term.	Moderate risk/issues; partially meets target. Less well accepted by older children and multiple suppositories required for higher doses. 4x daily dosing but short-term use.	2	The addition of other suppository strengths to EMLc would reduce the need for multiple suppository administration in older children (e.g., from ~ 6 years).
**Excipient safety**	Varies depending on formulation/PL. Contains commonly used suppository base excipients e.g., fats.	Low risk/no issues; meets target. Excipients generally have a suitable safety profile.	3	
**Administration considerations**	Suppositories are intended to be administered whole. No manipulation required, although need to be carefully inserted to avoid risk of premature expulsion and correct positioning in rectum.	Moderate risk/issues; partially meets target. Although no measuring device is required, some caregivers/patients may have difficulty with correct insertion, and some patients may experience discomfort. Suppository devices may be available. Higher doses would require multiple	2	May be more commonly used by HCPs rather than in domiciliary settings.

Attribute	Result	Comparison to Target and Risk Assessment	Score	Additional considerations/information
		suppositories to be administered.		
**Stability, storage conditions and primary packaging material**	Shelf life various from 2-3 years. Storage conditions- do not store above 30°C, do not store above 25°C, store away from heat and moisture. Primary pack is blisters.	Meets target/low risk - likely sufficient shelf life and product easily transported (generally light weight).	3	Some suppository bases may be more labile to heat and moisture.
**Registration status**	Numerous licences available, including in USA, UK, Europe, Australia- generic and branded. Other strength products also available in some markets (60, 80, 120, 125, 250, 325, 500mg). Posology appears to vary globally.	Meets target/low risk. Approved by stringent regulatory authorities.	3	

Table S4. Evaluation of Clofazimine (soft-gel capsules, 50 mg and 100 mg).

Attribute	Result	Comparison to Target and Risk Assessment	Score	Considerations
**Target population (age)**	**WHO recommendations:** (a) TB (section 6.2.5): MDR/RR-TB, 2-5 mg/kg for patients under 15 years, down to 5-6 kg (1 alt days); (b) Leprosy (section 6.2.4) Multibacillary leprosy (10-14 years), 3x50 mg Cfz (plus rifampicin and dapsone) day 1, 1x50 mg Cfz every other day, days 2-28. For children younger than 10y, 100mg once a month, 50 mg twice weekly)<br><br>**Indications:** UK: not found; Australia: not found; USFDA, discontinued (not reported); not found in EMA. Only available under special access programmes	Moderate risk/issues; partially meets target. WHO recommendations include younger children, but product is only available through special access programmes, it is not registered by any of the SRA reviewed.	2	
**Dose and dose flexibility**	Limited dose flexibility as the lowest strength is 50 mg. Dosing requires administration on alternate days (every other day or even less frequently for multibacillary leprosy for children <10y). This is acceptable due to the drug's long elimination half-life. Manipulating capsules to administer the contents is not possible, given that they are soft-gel capsules.	Moderate risk/issues; partially meets target. Dosing with available strengths requires administration on alternate days for children younger than 10 years.	2	Dosing on alternate days is acceptable due to the drug's long elimination half-life.
**Patient acceptability 0-5y**	Poor acceptability for younger children and those unable to swallow solid oral dosage forms. Dosing for children <10y is on alternate days (twice weekly, not frequent). Capsules are small (round 7 mm) and acceptable for patients aged from approximately 6 years.	High risk/issues; does not meet target for very young children and those unable to swallow capsules	1	
**Patient acceptability 6-12y**		Low risk/no issues; meets target, assuming the patient can swallow the capsule. Twice weekly dosing is acceptable and does not affect exposure, given clofazimine's long half-life.	3	
**Excipient safety**	Excipients in capsules include propylene glycol, parabens, flavouring agents	Moderate risk/issues; partially meets target. Contains 1 or 2 excipients of potential concern.	2	
**Administration considerations**	Capsules are intended to be swallowed whole. No manipulation required. However, soft gel capsules cannot be opened or dissolved in water and so young children are dosed every second or third day depending on body weight and the available formulation. .	Low risk/no issues; meets target as no manipulation required. However, some patients may have difficulty in swallowing the capsules whole. Dosing on alternate days permitted for young children requiring a lower dose. t	3	Dosing on alternate days is acceptable given the drug long half-life. There may be difficulties in administering the drug product to

Attribute	Result	Comparison to Target and Risk Assessment	Score	Considerations
				young children.
**Stability, storage conditions and primary packaging material**	Shelf life of 60 months. Do not store above 25˚C (77˚F) and protect from moisture. Preparation is supplied in a humidity-resistant container.	Moderate risk/issues; partially meets target. Shelf life acceptable but product requires protection from moisture.	2	
**Registration status**	Product not registered for leprosy nor for TB by any SRA. Branded name LAMPRENE (Novartis), available under special access (EAP) for NTM (https://www.clinicaltrials.gov/ct2/show/NCT04334070).	High risk/issues; does not meet target. Product not registered by any SRA for leprosy nor TB. Only available under special access conditions.	1	

Additional considerations: Clofazimine may cause orange-pink to brownish-black discoloration of the skin within a few weeks after you start using it. Because of the skin discoloration, some patients may experience depression.

Table S5. Evaluation of Clofazimine (tablets, 50 mg and 100 mg).

Attribute	Result	Comparison to Target and Risk Assessment	Score	Considerations
**Target population (age)**	**<u>WHO recommendations:</u>** (a) TB (section 6.2.5): MDR/RR-TB, 2-5 mg/kg for patients under 15 years, down to 5-6 kg (1 alt days); (b) Leprosy (section 6.2.4) Multibacillary leprosy (10-14 years), 3x50 mg Cfz (plus rifampicin and dapsone) day 1, 1x50 mg Cfz every other day, days 2-28. For children younger than 10y, 100 mg once a month, 50 mg twice weekly) **<u>Indications:</u>** UK: not found; Australia: not found; USFDA, discontinued (not reported); not found in EMA. Only available under special access programmes	Moderate risk/issues; partially meets target. WHO recommendations include younger children, but product is only available through special access programmes, it is not registered by any of the SRA reviewed.	2	
**Dose and dose flexibility**	Tablets are not scored or dispersible. Limited dose flexibility in increments of 50 mg or 100 mg. Dosing requires administration on alternate days (every other day or even less frequently for multibacillary leprosy for children <10y). This is acceptable due to the drug's long elimination half-life.	Moderate risk/issues; partially meets target. Limited dose flexibility. Dosing with available strengths requires administration on alternate days for children younger than 10 years.	2	Dosing on alternate days is acceptable due to the drug's long elimination half-life.
**Patient acceptability 0-5y**	Poor acceptability for younger children and those unable to swallow tablets. Dosing for children <10y is on alternate days (twice weekly, not frequent). Acceptable for patients able to swallow tablets.	High risk/issues; does not meet target for young children and those unable to swallow tablets.	1	
**Patient acceptability 6-12y**		Low risk/no issues; meets target assuming patient can swallow the tablet. Twice weekly dosing is permitted.	3	
**Excipients safety**	Excipients in tablets include polyoxyl hydrogenated castor oil and betadex (cyclodextrin).	Moderate risk/issues; partially meets target. Contains 1 or 2 excipients of potential concern, particularly for very young children.	2	Polyoxyl hydrogenated castor oil and cyclodextrins can cause stomach upset and diarrhoea.
**Administration considerations**	Tablets are intended to be swallowed whole. No manipulation required. Tablets are not dispersible and so young children are dosed every second or third day depending on body weight and the available formulation.	Low risk/no issues; meets target as no manipulation required. However, some patients may have difficulty in swallowing the tablets whole. Dosing on alternate days permitted for young children requiring a lower dose.	3	Dosing on alternate days is acceptable given the drug long half-life. There may be difficulties in administerin

Attribute	Result	Comparison to Target and Risk Assessment	Score	Considerations
				g the drug product to young children.
**Stability, storage conditions and primary packaging material**	Shelf life 24 months. Do not store above 30°C, protect from light. Avoid excursions above 30ºC.	Low risk/no issues; meets target.	3	
**Registration status**	Product not registered for leprosy nor for TB by any SRA. Branded name LAMPRENE (Novartis), available under special access (EAP) for NTM (https://www.clinicaltrials.gov/ct2/show/NCT04334070). Tablets (both strengths) are WHO Prequalified, but they are not as widely available as soft-gel capsules.	High risk/issues; does not meet target. Product not registered by any SRA for leprosy nor TB. Only available under special access conditions.	1	

Additional considerations: Clofazimine may cause orange-pink to brownish-black discoloration of the skin within a few weeks after you start using it. Because of the skin discoloration, some patients may experience depression.
